# Prognostic Significance of Cdc42 Expression in Colorectal Cancer and Its Concordance Between Primary Tumors and Matched Metastases: A Retrospective Observational Study

**DOI:** 10.3390/jcm15103848

**Published:** 2026-05-16

**Authors:** Oktay Halit Aktepe, Osman Butun, Olcay Kurtulan, Aziz Karaoglu, Suayib Yalcin

**Affiliations:** 1 Department of Medical Oncology, Dokuz Eylül University, Izmir 35330, Turkey; aziz.karaoglu@deu.edu.tr; 2 Department of Medical Oncology, Acibadem Izmir Kent Hospital, Izmir 35620, Turkey; osman.butun@acibadem.com; 3 Department of Pathology, Faculty of Medicine, Hacettepe University, Ankara 06230, Turkey; olcaykurtulan@gmail.com; 4 Department of Medical Oncology, Hacettepe University Cancer Institute, Ankara 06230, Turkey; syalcin@hacettepe.edu.tr

**Keywords:** colorectal cancer, Cdc42, prognostic biomarker, overall survival

## Abstract

**Background and Objectives:** Aberrant activation or overexpression of cell division cycle 42 (Cdc42) has been demonstrated in various tumors; however, its prognostic relevance in colorectal cancer (CRC) remains insufficiently defined. Thus, we evaluated the prognostic impact of Cdc42 expression in patients with CRC. In a paired primary–metastasis subset, we also assessed the concordance of Cdc42 expression between primary tumors and matched metastatic tissues. **Materials and Methods:** Cdc42 expression was assessed by immunohistochemistry in patients with colorectal cancer who underwent colectomy for curative or palliative purposes between January 2009 and January 2019. Cdc42 expression was quantified as a staining index and then dichotomized into low- and high-Cdc42 groups using a median cutoff value of six. Overall survival (OS) was analyzed by Kaplan–Meier curves with log-rank testing, and independent prognostic factors were assessed using Cox proportional hazard models. **Results:** The study included 94 patients (median age, 60 years) with a median follow-up of 88.4 months. High Cdc42 expression was significantly associated with Kirsten rat sarcoma viral oncogene homolog (KRAS) wild-type status (*p* = 0.001), lymph node metastasis (*p* = 0.039), and perineural invasion (*p* = 0.021). Patients with high Cdc42 expression had significantly poorer OS than those with low expression (median OS: 48.5 months, 95% confidence interval [CI]: 33.3–63.7 vs. 114.4 months, 95% CI: 24.0–204.9; *p* = 0.003). In multivariable Cox regression, high Cdc42 expression remained an independent predictor of worse OS (hazard ratio [HR]: 2.365, 95% CI: 1.336–4.184; *p* = 0.003), together with advanced stage and moderate-to-poor differentiation. In the paired primary–metastasis subset, Cdc42 expression in primary tumors correlated positively with that in matched metastases (Spearman ρ = 0.416, *p* = 0.016), whereas no overall directional shift between paired primary and metastatic samples was observed (Wilcoxon signed-rank test: Z = 0.423, *p* = 0.672). **Conclusions:** High Cdc42 expression may serve as an adverse prognostic marker in CRC. Cdc42 shows moderate concordance between primary tumors and matched metastases without a consistent directional shift.

## 1. Introduction

Colorectal cancer (CRC) remains one of the leading causes of cancer-related morbidity and mortality worldwide [1]. Despite advancements in early detection and treatment options [2,3,4,5,6], the heterogeneous nature of CRC is often associated with remarkable variations in patient outcomes [7,8]. Therefore, identifying reliable molecular biomarkers is crucial for better prognostication, providing enhanced survival in CRC.

Aberrant expression or activation of cell division cycle 42 (Cdc42), a member of the Rho family of GTPases, has been implicated in the progression, invasion, and metastatic potential of several malignancies, including CRC [9,10,11,12,13]. The relationship between Cdc42 and various oncogenic processes, including cytoskeletal reorganization, cell motility, proliferation, and polarity, has been established [14], contributing to tumor invasiveness and metastasis. Furthermore, Cdc42 may contribute to tumor microenvironment (TME) remodeling by promoting regulatory T-cell (Treg)-mediated immunosuppression and impairing antitumor immune responses [15]. It has been demonstrated that increased Cdc42 expression correlated with poorer survival in various cancers, including breast cancer, testicular cancer, esophageal cancer, and hepatocellular carcinoma [16,17,18,19]. Particularly, it contributes to the malignant phenotype of CRC by facilitating angiogenesis, promoting epithelial-to-mesenchymal transition, and regulating immune evasion [13,20,21]. These associations highlight the importance of understanding the role of Cdc42 in CRC, not only as a prognostic variable but also as a potential therapeutic target. However, despite this accumulating evidence, the association of Cdc42 expression with clinicopathologic characteristics and survival outcomes of CRC has not been comprehensively investigated. Therefore, the present study aimed to investigate the prognostic significance of Cdc42 in CRC. We also evaluated the concordance of Cdc42 expression between primary tumors and matched metastatic tissues.

## 2. Materials and Methods

### 2.1. Patients and Study Design

This observational study was conducted to investigate the prognostic significance of Cdc42 in a retrospective cohort of patients who underwent colectomy for curative or palliative purposes at initial presentation between January 2009 and January 2019. Patients’ data were obtained from the electronic records of Hacettepe University (Ankara, Turkey), including demographics (age, gender), clinical characteristics (M stage, metastatic region sites), tumor pathological characteristics (mutational status, tumor location site, lymphovascular invasion [LVI], perineural invasion [PNI], T stage, and N stage), treatment modalities (surgery, chemotherapy, radiotherapy), and follow-up information. The patients were included if they met all the following inclusion criteria: (1) histopathologically confirmed CRC diagnosis, (2) available and adequate tissue sample in our center, and (3) clinical follow-up data. The patients who received neoadjuvant chemotherapy or radiotherapy or those with incomplete clinical records were excluded.

Primary tumor samples used for Cdc42 assessment were obtained from colectomy specimens collected either during curative intent surgery or palliative resection at initial presentation. Matched metastatic samples were obtained from surgically resected metastatic lesions. The paired primary–metastasis cohort included patients with different clinical courses: some patients initially underwent curative surgery for localized CRC and later developed metastatic recurrence requiring metastasectomy, whereas others presented with metastatic disease, underwent palliative colectomy, received systemic treatment, and subsequently underwent resection of metastatic lesions. Therefore, the interval between primary and metastatic tissue sampling, as well as prior treatment exposure before metastasectomy, was not uniform across patients. This heterogeneity should be considered when interpreting the concordance of Cdc42 expression between primary and metastatic tissues.

### 2.2. Immunohistochemistry

**Figure 1:**
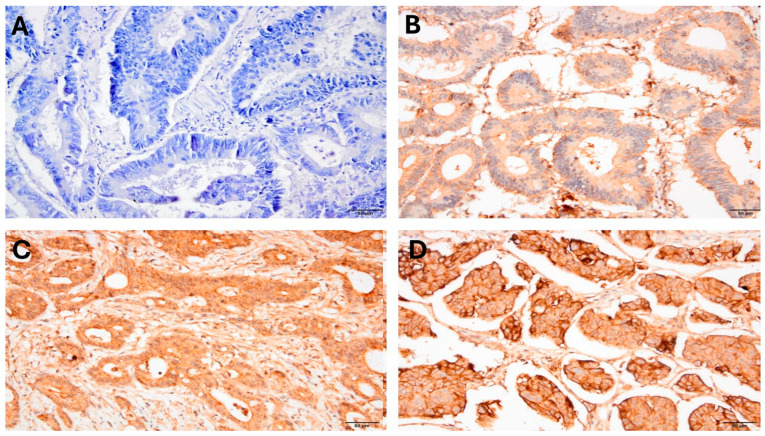
Representative images of Cdc42 expression in colorectal adenocarcinoma tissues stratified according to staining intensity at ×400 magnification: (**A**) negative staining, 0; (**B**) weak staining, 1+; (**C**) moderate staining, 2+; and (**D**) strong staining, 3+. Each panel illustrates the overall tumor cell staining pattern used for semiquantitative scoring.

To evaluate Cdc42 expression using immunohistochemistry, archival formalin-fixed paraffin-embedded (FFPE) blocks were obtained from the Department of Pathology at Hacettepe University. Representative tumor areas were marked on hematoxylin and eosin-stained slides, and the corresponding FFPE blocks were sectioned to a thickness of 4 µm. Following the manufacturer’s instructions, the tissue sections were deparaffinized in Bond Devax solution (Leica Microsystems) at 72° before being stained with a primary anti-Cdc42 antibody (Abcam, Cambridge, UK) using Leica Bond Autostainer. Two pathologists assessed the tumor cells’ membrane staining for Cdc42 expression in a blinded manner. We categorized tumors into four groups according to the estimated percentage of stained tumor cells with Cdc42: 1, 1–25%; 2, 26–50%; 3, 51–75%; and 4, >75%. As shown in Figure 1, using a four-tier method, the staining intensity was semiquantitatively grouped as follows: 0 negative, 1+ weak, 2+ moderate, and 3+ strong. The staining index was then measured as “the percentage score × staining intensity score”. As a result, the staining index ranged from 0 to 12.

### 2.3. Statistical Analyses

Categorical and continuous variables were presented as counts with percentages and median with interquartile range (IQR), respectively. Clinicopathologic characteristics were compared between Cdc42 expression groups using the Mann–Whitney U test for continuous data and the chi-square test or Fisher’s exact test for categorical data. Overall survival (OS) was defined as the time interval between the date of diagnosis and death from any cause or the last follow-up date. Survival estimates were evaluated in the Kaplan–Meier curves, and the log-rank was used for comparisons of survival rates. Patients were categorized into high- and low-Cdc42-expression groups according to the cohort’s median value of Cdc42 expression. Univariate Cox proportional hazard regression analyses were conducted to evaluate the impact of each clinicopathological variable, including Cdc42 expression levels, on OS. Multivariable Cox regression models were conducted with variables with a *p*-value of ≤0.2 in the univariate analyses. To assess the incremental prognostic value of Cdc42 expression, model fit was compared between a clinicopathologic Cox model including tumor stage and tumor differentiation and an extended model additionally including Cdc42 expression, using the change in −2 log likelihood. In the paired subset, Cdc42 expression between primary tumors and matched metastatic samples was compared using the Wilcoxon signed-rank test, and primary–metastasis concordance was assessed using Spearman’s rank correlation. All statistical analyses were performed using Statistical Package for Social Sciences (SPSS) version 27 (IBM Corp., Armonk, NY, USA), except for Kaplan–Meier curve generation, which was performed using R software (R Foundation for Statistical Computing, Vienna, Austria). A two-sided *p*-value < 0.05 was considered statistically significant.

## 3. Results

### 3.1. Patient Characteristics

The overall cohort included 94 patients with CRC, with a median age of 60 years (IQR: 57–64), who underwent colectomy for palliative or curative purposes. Utilizing the median cutoff value of six (IQR: 4–8) for Cdc42 expression, a total of 54 patients (57.4%) showed a Cdc42 expression ≥ median, regarded as the high-expression group, whereas 40 patients (42.6%) exhibited a Cdc42 expression < median, regarded as the low-expression group. The characteristics of all patients and stratified groups according to Cdc42 expression level are presented in Table 1. Within the cohort, there were 50 males (53.2%) and 44 females (46.8%). More than half of the patients had Kirsten rat sarcoma viral oncogene homolog (KRAS) wild type (56.4%) and distant metastasis at initial diagnosis (57.4%). Tumors were mainly located on the left side (75.5%). Approximately half of the patients had moderate-to-poorly differentiated tumors (48.9%), and the majority of tumors had the presence of LVI (63.8%) and PNI (72.3%). Patients with high Cdc42 expression had a significantly higher proportion of tumors with lymph node metastasis, PNI, and KRAS wild-type status than those with low Cdc42 expression (*p* = 0.039; *p* = 0.021; *p* = 0.001, respectively). There was no gender-specific difference in Cdc42 expression level; the distribution of male and female patients was similar between the low- and high-Cdc42-expression groups (male/female: 52.5%/47.5% vs. 53.7%/46.3%, respectively; *p* = 0.908). Overall, patients had the microsatellite-stable (MSS) phenotype. A doublet chemotherapy backbone plus a biologic agent including bevacizumab (an anti-vascular endothelial growth factor [VEGF] antibody) or cetuximab and panitumumab (anti-epidermal growth factor receptor antibodies) was used for the treatment of initial or metachronously metastatic CRC patients.

### 3.2. Correlation of Cdc42 Expression Between Primary Tumor and Metastasis

The paired comparison of Cdc42 expression between primary tumors and matched metastatic sites was performed in 33 patients. The liver was the most common metastasectomy site (n = 24, 72.8%), followed by the ovary (n = 4, 12.1%), lung (n = 3, 9.1%), small intestine (n = 1, 3.0%), and brain (n = 1, 3.0%). Cdc42 expression did not differ between liver and non-liver metastases (*p* = 0.951). Cdc42 expression in primary tumors correlated positively with that in corresponding metastases (Spearman ρ = 0.416, *p* = 0.016). However, there was no overall paired difference between primary and metastatic samples (Wilcoxon signed-rank test: n = 33, Z = 0.423, *p* = 0.672). Compared with the primary tumor, Cdc42 expression was higher in the metastatic site in 18 patients (54.5%), unchanged in 3 patients (9.1%), and lower in 12 patients (36.4%).

### 3.3. Survival Outcomes

**Figure 2:**
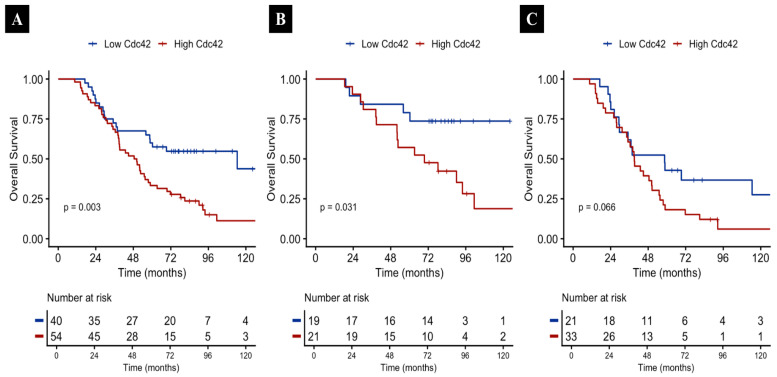
The Kaplan–Meier curves demonstrating estimated OS stratified according to Cdc42 expression level in all patients (**A**), patients with early-stage disease (**B**), and patients with advanced-stage disease (**C**).

A total of 64 patients died during the median follow-up time of 88.4 months (95% confidence interval [CI]: 81–95.9). The median OS was 57 months. The high-Cdc42-expression group had shorter median OS than the low-Cdc42-expression group (48.5 months, 95% CI: 33.3–63.7 vs. 114.4 months, 95% CI: 24–204.9, *p* = 0.003, Figure 2A). In patients with early-stage disease, median OS was shorter in the high-Cdc42-expression group than in the low-Cdc42-expression group (69.5 months, 95% CI: 32.6–106.4 vs. median not reached, *p* = 0.031, respectively, Figure 2B). In patients with advanced-stage disease, median OS was numerically shorter in the high-Cdc42-expression group, although the difference was not statistically significant (39.1 months, 95% CI: 31.8–46.4 vs. 58.5 months, 95% CI: 26–90.9, *p* = 0.066, respectively, Figure 2C).

As shown in Table 2, in univariate Cox regression analysis, the variables significantly associated with worse OS were moderate–poor differentiation (hazard ratio [HR]: 2.281; 95% CI: 1.372–3.790; *p* = 0.001), presence of PNI (HR: 2.141; 95% CI: 1.170–3.919; *p* = 0.014), advanced stage (HR: 2.465; 95% CI: 1.445–4.204; *p* = 0.001), and high Cdc42 expression status (HR: 2.229; 95% CI: 1.300–3.821; *p* = 0.004). The other potential variables of worse OS were male gender (HR: 1.413; 95% CI: 0.856–2.331; *p* = 0.176) and right-side location (HR: 1.617; 95% CI: 0.874–2.990; *p* = 0.126). All these variables were subsequently evaluated in multivariable Cox regression analysis. As shown in Table 3, multivariable analyses showed that high Cdc42 expression was an independent adverse prognostic variable for OS (HR: 2.365; 95% CI: 1.336–4.184; *p* = 0.003). The other identified adverse prognostic factors for OS were moderate–poor differentiation (HR: 2.687; 95% CI: 1.556–4.642; *p* < 0.001) and advanced stage (HR: 2.424; 95% CI: 1.370–4.286; *p* = 0.002).

To further assess the incremental prognostic value of Cdc42 expression, we compared a clinicopathologic Cox model including tumor stage and tumor differentiation with an extended model additionally including Cdc42 expression. Adding Cdc42 expression significantly improved model fit, with a reduction in −2 log likelihood (−2LL) from 490.442 to 477.850. The change in −2LL was 12.592 with 1 degree of freedom (*p* < 0.001). In the extended model, high Cdc42 expression remained independently associated with poorer OS (HR: 2.632, 95% CI: 1.502–4.612; *p* < 0.001), suggesting that Cdc42 expression provides additional prognostic information beyond tumor stage and differentiation.

## 4. Discussion

The present study demonstrates that high Cdc42 expression is associated with adverse clinicopathologic features and inferior OS in patients with CRC. Importantly, high Cdc42 expression remained an independent adverse prognostic variable in predicting OS after adjustment for established prognostic factors, including tumor stage and tumor differentiation. Moreover, the additional model comparison analysis supported the complementary prognostic contribution of Cdc42 expression beyond these conventional clinicopathologic variables. These findings suggest that Cdc42 expression should not be interpreted as a replacement for conventional clinicopathologic parameters but rather as a complementary biomarker that may reflect additional tumor biological aggressiveness. While tumor stage and differentiation represent established anatomic and histopathologic prognostic factors, Cdc42 expression may provide additional biological information related to tumor aggressiveness.

In the present cohort, high Cdc42 expression was significantly associated with lymph node metastasis, PNI, and KRAS wild-type status. These findings support the possible relationship between Cdc42 expression and more aggressive tumor behavior in CRC. Gómez Del Pulgar et al. reported that Cdc42 was overexpressed in approximately 60% of CRC samples and that its expression was significantly associated with tumor grade, a feature linked to more aggressive histology [22]. Consistent with this finding, we observed high Cdc42 expression in 57.4% of CRC cases. However, we also demonstrated significant associations between Cdc42 expression and tumor characteristics, including wild-type KRAS status, lymph node metastasis, and PNI. In line with these findings, Chen et al. reported that patients with lung adenocarcinoma expressing Cdc42 were more likely to have lymph node metastasis [23]. Additionally, to the best of our knowledge, this is the first study to report a positive correlation between Cdc42 expression in primary CRC tumors and matched metastatic tissues.

The relationship between Cdc42 expression and aggressive clinicopathologic features has also been reported in other malignant tumors. In cervical squamous cell carcinoma, Ma et al. showed that Cdc42 protein expression was significantly higher in tumor tissues than in normal cervical tissues and that higher Cdc42 expression was associated with more advanced clinical stage [24]. Similarly, the role of Cdc42 in gastric cancer further supports its relevance in tumor proliferation and invasion. Du et al. reported that Cdc42 knockdown inhibited the proliferation and invasion of gastric cancer cells, indicating that Cdc42-related signaling may contribute to aggressive tumor cell behavior [25]. In addition to Cdc42 itself, upstream regulatory pathways may further enhance Cdc42-mediated tumor progression. ArfGAP with SH3 domain, ankyrin repeat, and PH domain 1 (ASAP1) encodes an ADP-ribosylation factor GTPase-activating protein involved in cytoskeletal remodeling, integrin recycling, and cancer cell invasion [26,27,28]. More recently, Xie et al. showed that ASAP1 activates the IQ motif containing the GTPase-activating protein 1 (IQGAP1)/Cdc42 pathway to promote tumor progression and chemotherapy resistance in gastric cancer [29]. Taken together, these findings suggest that Cdc42-related pathways may be associated with aggressive clinicopathologic features, tumor cell invasion, and treatment resistance across different malignancies, supporting our observation that high Cdc42 expression is associated with lymph node metastasis, PNI, and poorer OS in CRC.

Angiogenesis is one of the most critical pathological processes for tumor progression, recurrence, and metastasis. Cdc42 is associated with endothelial cell adhesion, invasion, proliferation, and migration, all of which are essential for neovascularization [30,31]. It also modulates the angiogenic switch in the TME by regulating mammalian target of rapamycin and phosphoinositide 3-kinase signaling pathways [32,33]. Endothelial cell tubule formation is triggered by Cdc42-mediated signaling pathways [34], thereby contributing to the formation of a vascularized TME. Moreover, Cdc42 has also been shown to play a pivotal role in angiogenesis by regulating several morphogenetic mechanisms during angiogenic sprouting in vitro [35]. In CRC, Ma et al. further demonstrated that VEGF/neuropilin-1 (NRP1) engagement induces the subcellular relocation of Cdc42, promoting filopodia and invadopodia formation, directional migration, invasion, and metastatic behavior [20]. Importantly, this VEGF/NRP1-mediated Cdc42 relocation was associated with aggressive tumor behavior and poor prognosis in patients with CRC [20]. Although our study assessed total Cdc42 expression rather than its subcellular localization, these findings provide a biologically plausible explanation for the association between high Cdc42 expression and inferior OS observed in our cohort.

Cdc42 may also be involved in the immune sculpting of the TME. The ability of tumors to evade the destruction by immune attack is a crucial aspect of cancer progression [36]. It has been demonstrated that high Cdc42 expression is associated with alterations in the expression of several immune checkpoint molecules, including programmed death 1 and programmed death ligand 1 [15]. Treg cells in the TME suppress antitumor immune responses, particularly by inhibiting effector T-cell activity, including CD8+ T-cell function [37]. Cdc42 activity has also been implicated in the regulation of Treg stability and antitumor T-cell immunity, supporting a potential role for Cdc42-related signaling in shaping an immunosuppressive TME [38]. These findings suggest that Cdc42 may be related not only to tumor cell invasion and migration but also to immune evasion in the TME.

Additional experimental data also support the possible functional relevance of Cdc42 in CRC. In the study by Gómez Del Pulgar et al., overexpression of Cdc42 was suggested to downregulate inhibitor of DNA binding 4, a tumor suppressor protein (TSP), contributing to the development of CRC through an epigenetic mechanism [22]. Cdc42 expression also negatively correlates with the expression of CACNA2D2 [39], which functions as a TSP in several cancers [40]. Zins et al. analyzed the activity of AZA197, a selective inhibitor of Cdc42, in SW620 and HT-29 human CRC cells and showed that it downregulated the extracellular signal-regulated kinase and p21-activated kinase 1 signaling pathways, resulting in enhanced apoptosis and suppressed cell invasion, migration, and proliferation [41]. These experimental and translational data support our clinical observation that high Cdc42 expression is associated with poorer OS in CRC.

From a clinical perspective, our findings should be interpreted as hypothesis-generating rather than immediately practice-changing. Cdc42 expression may help refine prognostic assessment by identifying patients with biologically aggressive disease, but its incremental clinical value over tumor stage and differentiation should be confirmed in larger independent cohorts. Future studies should also evaluate whether compartment-specific Cdc42 expression, particularly subcellular relocation patterns such as VEGF/NRP1-mediated localization at the migrating cell front, provides stronger prognostic information than total Cdc42 expression. Moreover, future experimental and translational studies may clarify whether targeting Cdc42-related pathways could reduce tumor cell motility, invasion, angiogenesis, immune evasion, and metastatic dissemination in CRC.

While our study elucidates the significant relationship between high Cdc42 expression and worse OS in CRC patients, the major limitation of the present study was its retrospective nature. Secondly, the cohort sample size was relatively small, which may limit the generalizability of our results. Thirdly, this study primarily focused on the relationship between Cdc42 expression levels and survival outcomes, lacking functional assays to demonstrate the exact role of Cdc42 in CRC biology. Thus, the functional role of Cdc42 should be tested in future studies using in vitro and in vivo models. Fourthly, all patients in our study had an MSS phenotype; therefore, the prognostic significance of Cdc42 expression in patients with high microsatellite instability or mismatch repair-deficient CRC should be clarified in future studies. Fifthly, some potentially relevant clinical variables, including body mass index and history of other malignant diseases, were not consistently available in the retrospective medical records and therefore could not be included in the baseline clinicopathologic characteristics or adjusted for in the survival analyses. Finally, because Cdc42 expression was assessed only in FFPE tumor tissue samples, its expression in adjacent normal colon tissue could not be evaluated.

## 5. Conclusions

Our findings suggest that high Cdc42 expression is associated with adverse clinicopathologic features and poorer OS in patients with CRC. The prognostic and therapeutic relevance of Cdc42 expression should be evaluated in prospective studies to determine its potential role in individualized risk stratification and treatment strategies in CRC.

## Figures and Tables

**Table 1:** Baseline clinicopathologic patient characteristics of all patients and stratified groups based on Cdc42 expression status.

Cdc42 Expression				
Variable	All Patients (n = 94)	Low (<6) n = 40 (42.6%)	High (≥6) n = 54 (57.4%)	*p*-Value
Gender				0.908
Male	50 (53.2%)	21 (52.5%)	29 (53.7%)	
Female	44 (46.8%)	19 (47.5%)	25 (46.3%)	
Age				0.639
<65	73 (77.7%)	32 (80%)	41 (75.9%)	
≥65	21 (22.3%)	8 (20%)	13 (24.1%)	
T stage T2-3	47 (50%)	18 (45%)	29 (53.7%)	0.404
T4	47 (50%)	22 (55%)	25 (46.3%)	
Lymph node status				0.039
N0	25 (26.6%)	15 (37.5%)	10 (18.5%)	
N1-2-3	69 (73.4%)	25 (62.5%)	44 (81.5%)	
KRAS mutation status				0.001
Wild	53 (56.4%)	15 (37.5%)	38 (70.4%)	
Mutant	41 (43.6%)	25 (62.5%)	16 (29.6%)	
M0	40 (42.6%)	19 (47.5%)	21 (38.9%)	0.404
M1	54 (57.4%)	21 (52.5%)	33 (61.1%)	
LVI				0.817
Absent	34 (36.2%)	15 (37.5%)	19 (35.2%)	
Present	60 (63.8%)	25 (62.5%)	35 (64.8%)	
PNI				0.021
Absent	26 (27.7%)	16 (40%)	10 (18.5%)	
Present	68 (72.3%)	24 (60%)	44 (81.5%)	
Tumor location				0.918
Left	71 (75.5%)	30 (75%)	41 (75.9%)	
Right	23 (24.5%)	10 (25%)	13 (24.1%)	
Tumor differentiation				0.859
Good	48 (51.1%)	20 (50%)	28 (51.9%)	
Moderate–poor	46 (48.9%)	20 (50%)	26 (48.1%)	

**Abbreviations:** Cdc42: cell division cycle 42; KRAS: Kirsten rat sarcoma viral oncogene homolog; LVI: lymphovascular invasion; PNI: perineural invasion.

**Table 2:** Univariate Cox proportional hazard analysis of prognostic factors for OS.

Variable	HR	95% CI		*p*-Value
		Lower	Upper	
Age (≥65 vs. <65)	1.008	0.565	1.799	0.978
Gender (male vs. female)	1.413	0.856	2.331	0.176
Tumor differentiation (moderate–poor vs. good)	2.281	1.372	3.790	0.001
Tumor location (right vs. left)	1.617	0.874	2.990	0.126
LVI (present vs. absent)	1.409	0.833	2.381	0.201
PNI (present vs. absent)	2.141	1.170	3.919	0.014
Tumor stage (metastatic vs. early)	2.465	1.445	4.204	0.001
Cdc42 expression (high vs. low)	2.229	1.300	3.821	0.004

**Abbreviations:** Cdc42: cell division cycle 42; CI: confidence interval; HR: hazard ratio; LVI: lymphovascular invasion; OS: overall survival; PNI: perineural invasion.

**Table 3:** Multivariable Cox proportional hazard analysis of independent prognostic factors for OS.

Variable	HR	95% CI		*p*-Value
		Lower	Upper	
Gender (male vs. female)	1.685	0.973	2.918	0.063
Tumor differentiation (moderate–poor vs. good)	2.687	1.556	4.642	<0.001
Tumor location (right vs. left)	1.175	0.611	2.257	0.629
PNI (present vs. absent)	1.363	0.681	2.730	0.382
Tumor stage (metastatic vs. early)	2.424	1.370	4.286	0.002
Cdc42 expression (high vs. low)	2.365	1.336	4.184	0.003

**Abbreviations:** Cdc42: cell division cycle 42; CI: confidence interval; HR: hazard ratio; OS: overall survival; PNI: perineural invasion.

## Data Availability

Data are available upon reasonable request.

## Abbreviations

The following abbreviations are used in this manuscript:

ASAP1	ArfGAP with SH3 domain, ankyrin repeat and PH domain 1
Cdc42	cell division cycle 42
CI	confidence interval
CRC	colorectal cancer
FFPE	formalin-fixed paraffin-embedded
HR	hazard ratio
IQGAP1	IQ motif containing GTPase-activating protein 1
IQR	interquartile range
KRAS	Kirsten rat sarcoma viral oncogene homolog
LVI	lymphovascular invasion
NRP1	neuropilin-1
MSS	microsatellite stable
OS	overall survival
PNI	perineural invasion
SPSS	Statistical Package for Social Sciences
TME	tumor microenvironment
Treg	regulatory T cell
TSP	tumor suppressor protein
VEGF	vascular endothelial growth factor
